# Construction of Phylogenetic Relationships Based on 8-mer Spectra Distribution Characteristics of Vertebrate Whole Genome Sequences

**DOI:** 10.3390/genes17010039

**Published:** 2025-12-31

**Authors:** Zhenhua Yang, Li Wang, Guojun Liu, Dongsheng Yu, Xiangjun Cui

**Affiliations:** 1 School of Economics and Management, Inner Mongolia University of Science & Technology, Baotou 014010, China; 2 Faculty of Chemistry, Baotou Teachers’ College, Baotou 014030, China; 3 School of Life Science and Technology, Inner Mongolia University of Science & Technology, Baotou 014010, China

**Keywords:** vertebrate genomes, distribution characteristics of 8-mer spectra, evolutionary features, phylogenetic relationship

## Abstract

**Background/Objectives**: With advances in sequencing technology, whole genome sequences have become a valuable resource for deciphering species evolution. However, efficiently extracting phylogenetic information from such data remains a major challenge. Traditional multiple sequence alignment methods are computationally intensive and perform poorly for distantly related species, while k-mer analysis offers a new direction for efficiently capturing genomic composition and evolutionary signatures. **Methods**: Feature extraction based on 8-mer spectra from 16 XYi subsets. **Results**: This study found that the distribution characteristics of whole genome sequences 8-mer spectra are closely related to species evolution. Building on this, we developed a dual-feature strategy for genome-scale phylogenetics. The strategy incorporates two distinct feature types: (a) 186 class-level phylogenetic features (comprising 93 for separability and 93 for conservatism), identified from 8-mer spectrum distributions of 16 XYi subsets, which capture macroevolutionary patterns; and (b) order-level phylogenetic features, designated as rank information, which are generated by ranking all 65,536 8-mers by frequency based on the CGi subset’s long-tail distribution and thereby capture microevolutionary patterns. Validation across vertebrate genomes confirmed that the class-level features establish the phylogenetic backbone, whereas the order-level features enable finer-resolution discrimination at the ordinal level. **Conclusions**: This study proposes a new method for constructing phylogenetic relationships at the genomic level.

## 1. Introduction

Mining the information in nucleotide sequences is one of the most fundamental aspects of bioinformatics. For large-scale genomic studies, datasets from initiatives such as the 1000 Genomes Project [1], the Encyclopedia of DNA Elements (ENCODE) [2], Translation by the Earth Biogenome Project (EBP) [3], and the Integrative Human Microbiome Project [4] serve as invaluable resources for genome research. Nevertheless, the enormous scale of data produced by these initiatives creates formidable obstacles for computational analysis. K-mers analysis has evolved into a paradigm-shifting technique for biological sequence analysis, addressing critical challenges posed by the exponential growth of genomic data.

In 1986, Brendel [5] first proposed the k-mers method, utilizing the non-random distribution features of k-mer (k = 3, 4, 5) frequencies to uncover functional and evolutionary relationships in genomic sequences. Gradually, k-mer analysis became widely adopted in genomics and proteomics applications. In genomic sequence analysis, k-mer frequencies have emerged as a powerful approach for functional annotation of regulatory elements directly from DNA sequences, such as enhancers [6], promoters [7], and insulators [8]. Recent methodological advances have further enhanced prediction accuracy through the integration of k-mer frequencies with machine learning techniques, establishing a new method for computationally identifying regulatory genomics elements [9,10,11]. Based on the specific binding between k-mers and proteins, researchers can predict nucleosome occupancy [12], determine RNA folding patterns [13], and explore the genomic features underlying high-occupancy target (HOT) regions [14,15]. The differential usage of k-mers has been effectively applied to sequence comparison [16,17], genome assembly [18,19], and evolutionary pattern identification across species [20,21].

In metagenomic studies, k-mer frequencies serve as essential features for taxonomic classification [22,23] and functional annotation [24]. Moreover, within biomedical research, numerous institutions now perform high-throughput sequencing of patient cohorts. This enables researchers to employ k-mer approaches for comprehensive analysis of disease-associated genomic data, thereby advancing the implementation of precision medicine. Notable applications include Noninvasive Prenatal Screening for Fetal Aneuploidies [25], investigating the association between microRNAs/circRNAs and complex diseases [26,27], pathogen and cancer detection [28], vaccine development [29], and cancer therapeutics [30,31].

Traditionally, phylogenetic tree construction has been based on multiple sequence alignments of conserved proteins or homologous genes. Commonly used sequence alignment-based algorithms, such as BLAST [32], Muscle [33], and ClustalW [34]. In recent years, genome sequencing technology has developed rapidly, leading to a substantial accumulation of whole-genome sequence data in the public archives. However, alignment-based algorithms for whole genome sequence analysis exhibit significant computational limitations, particularly regarding their time complexity and memory demands [35]. To overcome the computational bottlenecks of alignment-based approaches, researchers have proposed various alignment-free algorithms for sequence comparison. Among these methods, k-mer frequency analysis has emerged as a fundamental technique for constructing whole genome phylogenetic relationships, with the Feature Frequency Profile (FFP) [36] method being one of the most prominent approaches. The FFP method has been applied to construct phylogenetic relationships among prokaryotic and multicellular eukaryotic genome sequences, such as bacteria [37], fungi [38], plants [39], and mammals [40]. In recent years, alignment-free methods utilizing k-mer frequencies in comparative genomics have been constantly updated. At present, the more common methods include kMetaShot [41], kmdiff [42], and CMash [43].

Research on the k-mer spectrum distributions of genomic sequences and their relationship with genome evolution has achieved substantial advancements. The k-mer spectrum distributions serve as a unique signature reflecting species-specific genomic composition and evolutionary characteristics. At first, by analyzing the 6-mer spectrum distribution of nine species, researchers identified two key results [44]: (a) Marked disparities exist between the 6-mer spectrum distributions of genomic sequences and random sequences. In random sequences, 6-mers exhibit nearly equal occurrence probabilities, whereas genomic sequences demonstrate 6-mer usage bias. (b) As species evolve from primitive to complex, their genomic 6-mer spectrum distribution transitions from a unimodal to a multimodal pattern. Next, Chor [45] studied the k-mer spectra distribution characteristics of more than 100 species genomes. The research results revealed that archaeal, bacterial, and fish species exhibit unimodal k-mer spectrum distributions, whereas all mammals and tetrapods in non-mammalian species exhibit multimodal k-mer spectrum distributions. This finding further demonstrates the close correlation between k-mer spectrum distributions and the evolution of genome sequences. Recently, some researchers have focused on the k-mer spectra characteristics of each functional fragment in the genome sequence. For example, k-mer spectra as a kernel function can reveal evolutionary differences in exon, intron, and CpG island sequences in mammal genomes [46], and k-mer spectra combining Hi-C datasets predict DNA breakpoint regions [47].

We recognize that the k-mer spectra of genomic sequences serve as a window, enabling us to glimpse into the composition and evolutionary features of genome sequences. Therefore, investigating the intrinsic law of the k-mer spectra of genome sequences is critical for decoding genomic information. In this study, we systematically investigate distribution characteristics of 8-mer spectra to uncover novel evolutionary features in vertebrate genomic sequences. Building on this foundation, we develop an innovative computational approach to capture evolutionary features for sequence comparison.

## 2. Materials and Methods

### 2.1. Vertebrate Genome Data

The genome sequences and corresponding annotation data were obtained from UCSC (http://genome.ucsc.edu/) and NCBI (https://www.ncbi.nlm.nih.gov/), encompassing a total of 118 selected species. X chromosomes were excluded from genome analyses. Species genome taxonomy is presented in Table 1 (see Supplementary Table S1 for details).

### 2.2. 8-mer Spectrum Distribution of Genome Sequence

For a given DNA sequence, total 8-mer occurrence frequencies were calculated by using 8 bp as the window and 1 bp as a sliding step along the sequence. The relative motif number (*RMN*) is defined as follows:

(1) $$RMN=\frac{N_{i}}{4^{8}}$$

N_i_ is the number of 8-mers with frequency *i*. The distribution constructed with 8-mer frequency i as the *x*-axis and relative motif number (*RMN*) as the *y*-axis is defined as the 8-mer spectrum distribution (abbreviated as 8-mer spectra).

### 2.3. Random Center

The random center is the average frequency of the total 8-mers. We computed the occurrence frequencies of a total of 8-mers (65,536) in each genome sequence, where $N_{i}$ represents the frequency of the *i*th 8-mer. The random center ($\bar{N}$) is defined as follows:

(2) $$\bar{N}=\frac{\sum_{i=1}^{4^{8}}N_{i}}{4^{8}}$$

### 2.4. XY Dinucleotide Classification Method

Analyzing the relationship between 8-mer occurrence frequencies and compositional characteristics, we classified the total 8-mers into distinct subsets. For this purpose, we proposed the XY dinucleotide classification method, defined as follows:

The 8-mer containing none XY dinucleotide is denoted as XY0 8-mer, containing one XY dinucleotide is denoted as XY1 8-mer, and containing two or more XY dinucleotides is denoted as XY2 8-mer.

The total 8-mer set consists of 4^8^ = 65,536. When X ≠ Y, there are 40,545 XY0 8-mers, 21,468 XY1 8-mers, and 3523 XY0 8-mers. When X = Y, there are 44,631 XY0 8-mers, 14,931 XY1 8-mers and 5974 XY1 6-mers. Thus, we obtained spectra for 48 XYi (X, Y ∈ {A, T, C, G}; i = 0, 1, 2) 8-mer subsets.

### 2.5. XYZ Trinucleotide Classification Method

We proposed a systematic method to classify a total of 9-mers according to the content of XYZ trinucleotides (where X, Y, Z ∈ {A, T, C, G}). Classification is performed as follows:

For a given 9-mer, if it contains zero XYZ trinucleotide, it is classified as XYZ0. If it contains one XYZ trinucleotide, it is classified as XYZ1. If it contains two or more XYZ trinucleotides, it is classified as XYZ2. Since there are 64 possible XYZ trinucleotide combinations, this classification method produces 192 distinct 9-mer subsets.

Theoretically, the total 9-mer set consists of 4^9^ = 262,144 motifs. When X = Y = Z, the number of XYZ0 9-mer subsets is 239,868, the number of XYZ1 9-mer subsets is 17,226, and the number of XYZ2 9-mer subsets is 5050. When X ≠ Y ≠ Z, X = Y ≠ Z, or X ≠ Y = Z, the number of XYZ0 9-mer subsets is 234,111, the number of XYZ1 9-mer subsets is 27,395, and the number of XYZ2 9-mer subsets is 638. When X = Z ≠ Y, the number of XYZ0 9-mer subsets is 235,320, the number of XYZ1 9-mer subsets is 25,047, and the number of XYZ2 9-mer subsets is 1777.

### 2.6. Separability and Conservatism Features

In order to study the positional difference and the conservative degree of CGi/XYi_CGj 8-mer spectra in a genome sequence and compare the characteristics among different genomes, we defined the separability and the conservatism values.

(1) Separability

(3) $$\delta_{i}=\log_{2}\frac{\bar{x}}{{\bar{x}}_{i}}$$

where $\bar{x}$ is the random center and $\bar{x_{i}}$ is the average frequency of the *i*th 8-mer subset. The parameter $\delta_{i}$ quantifies the spectrum separation degree between the *i*th subset and the random center. Specifically, if $\delta_{i}$ > 1, it indicates that the *i*th 8-mer subset spectrum is distributed at the low-frequency end and deviates from the random center. If $\delta_{i}$ = 1, it suggests that the *i*th 8-mer subset spectrum is distributed similarly to the random center.

(2) Conservatism

(4) $$\beta_{i}=\log_{2}\frac{SD}{{SD}_{i}}$$

where *SD* is the standard deviation of the total 8-mer spectrum and ${SD}_{i}$ is the standard deviation of the *i*th subset spectrum. The $\beta_{i}$ represents the conservatism degree of the *i*th subset spectrum relative to the total 8-mer spectrum.

### 2.7. Constructing Phylogenetic Relationships

(a) Constructing Phylogenies Using Class-Level Features

We used the separability and conservatism of CGi/XYi_CGj subsets’ spectra as the characteristic parameters to construct a phylogenetic tree. The evolutionary distance $D_{nm}$ between two species’ genomes is defined as follows:

(5) $$D_{nm}=\sum_{k=1}^{93}\sqrt{{\left(\delta_{nk}-\delta_{mk}\right)}^{2}+{\left(\beta_{nk}-\beta_{mk}\right)}^{2}}$$

where $\delta_{nk}$ represents the separability value of CGi/XYi_CGi subset spectra in the *n*th species genome, and $\delta_{mk}$ represents the separability value of CGi/XYi_CGj subset spectra in the *m*th species genome. $\beta_{nk}$ represents the conservatism value of the CGi/XYi_CGj subset spectra in the *n*th species genome, and $\beta_{mk}$ represents the conservatism value of the CGi/XYi_CGj subset spectra in the *m*th species genome. According to the distance matrix (*D*), a phylogenetic tree was constructed using the neighbor-joining method by Mega12 software.

(b) Constructing Phylogenies Using Order-Level Features

The evolutionary distance $W_{nm}$ between two species’ genomes is defined as follows:

(6) $$W_{nm}=\sum_{k=1}^{65,536}\sqrt{{\left(R_{nk}-R_{mk}\right)}^{2}}$$

where $R_{nk}$ represents the Rank value of the *k*th 8-mer in the *n*th species genome, and $R_{mk}$ represents the Rank value of the *k*th 8-mer in the *m*th species genome. According to the distance matrix (*W*), a phylogenetic tree was constructed using the neighbor-joining method by the Mega12 software.

The advantages of our method are primarily reflected in the following two aspects: (a) Standardized processing: The ranking method eliminates the direct influence of genome size on analytical results, ensuring comparability between genomes of different scales. (b) Pattern-driven comparison: Genomes are treated as a whole and compared based on their 8-mer composition and spectra distribution characteristics, rather than relying on traditional sequence alignment and base substitution analysis. This approach avoids dependence on the correction of evolutionary distances and enhances applicability across distantly related species and at whole-genome scales.

## 3. Results

### 3.1. Spectrum Distribution of Non-CG Class Subsets

The k-mer spectrum serves as a fundamental method for characterizing genomic sequence information. In eukaryotic genomes, the *k*-value is selected as 8. The reasons are as follows: (a) To select the appropriate length of k-mers, we analyzed the k-mer spectrum distributions across a range of *k-values* (k = 7 to 12) using the whole genome sequence of *Homo sapiens*. It is found that from k ≥ 8, the k-mer spectrum distributions gradually tend to be stable, and the distribution characteristics of tri-modal have appeared. Still, from k > 12, the distribution patterns of tri-modal have disappeared. (b) The length of eukaryotic transcription factor binding sites is typically approximately eight base pairs. (c) To ensure statistically significant representation of rare k-mer frequencies in DNA sequence, Chor used *k*-values that are $k=\left\lfloor 0.7{log}_{4}^{l}\right\rfloor$, where L is the length of the DNA sequence [44]. For *Saccharomyces cerevisiae*, which possesses a relatively small eukaryotic genome, this calculation yields k ≈ 8.9. Given these considerations and to maintain generality across eukaryotic genomes, we ultimately selected k = 8 for our analyses.

**Figure 1:**
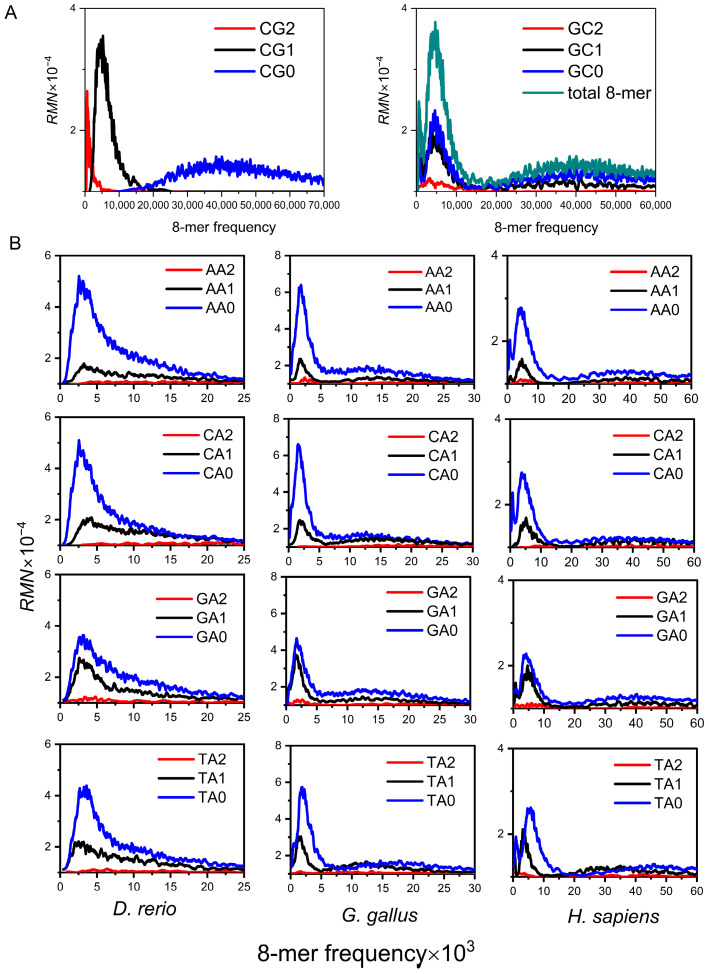
Spectra of 8-mer subsets from genome sequences. (**A**) The 8-mer spectrum distribution of CG and GC subsets in the human genome. (**B**) The 8-mer spectrum distribution of non-CG class subsets (illustrated by the AA, CA, GA, and TA subsets) for the three representative species’ genome sequences.

Our initial analysis of the 8-mer spectrum distribution across 16 XY dinucleotide classifications in diverse species revealed that only the CG classification exhibits the following three properties: (a) Evolutionary independence, the 8-mer spectra of CG0, CG1, and CG2 subsets form an independent unimodal distribution, which corresponds to the three peaks of the total 8-mer spectra (Figure 1A). (b) Evolutionary separability, compared with the random center, the frequencies of CG1 and CG2 8-mer subsets are much lower than the random center, and the spectrum distribution of the CG0 subset is spread around the random center (Figure 1A). (c) Evolutionary conservatism, the 8-mer spectra of the CG1 and CG2 subsets are much narrower than that of the CG0 subset, which indicates the usage of the CG1 and CG2 8-mers is conservative (Figure 1A). We identified these distinctive spectrum distribution characteristics, which we refer to as the CG-independent selection phenomenon in genome sequences. This phenomenon indicates that the distribution characteristics of 8-mer spectra in the three CGi subsets are closely related to species evolution [48,49].

Previous studies only analyzed the spectrum distribution characteristics of three CGi subsets [48], whereas to comprehensively represent the evolutionary information of the genome, it is necessary to cover the distribution characteristics of all 16 XYi subsets. Here, we further analyzed the distribution characteristics of 8-mer spectra in the other 15 XYi subsets for vertebrate genome sequences. For the convenience of expression, we refer to the other 15 XYi 8-mer subsets as non-CG class subsets. Analysis of 8-mer spectra in non-CG class subsets across diverse vertebrate genomes revealed distinct evolutionary features (Figure 1B). Unimodal distribution is observed in fishes (e.g., *Danio rerio*). Bimodal distribution is observed in Lepidosauria and birds (e.g., *gallus*). Tri-modal distribution is observed in mammals, including other mammals, rodents, and primates (e.g., *H. sapiens*). Within a given species, the 8-mer spectrum distributions of non-CG class subsets are highly consistent. Therefore, Figure 1B only displays the distributions for the AAi, CAi, GAi, and TAi subsets. As vertebrate genomes evolved increasingly complex, the 8-mer spectrum distributions of non-CG class subsets show the same transition process from unimodal to multimodal as the total 8-mer spectra. We postulated that the multimodal distribution characteristics of 8-mer spectra in non-CG class subsets must contain more information about the composition and evolution of the genome sequence.

According to statistical theory, if a population consists of sample units with identical properties, the distribution characteristics of the sample should be an unimodal distribution [50]. If a population consists of sample units with heterogeneous properties, the distribution characteristics of the sample should be multimodal [50]. The research results indicate that the 8-mer spectrum distributions of non-CG class subsets in mammals exhibit tri-modal, which implies these 8-mers come from three different populations. The 8-mer spectra of the non-CG class subsets (fishes) are unimodal. Do these 8-mers still come from three different populations? Only by strictly separating these three motifs can the mechanism be further revealed.

### 3.2. Class-Level Phylogenetic Features

To systematically characterize the compositional features of non-CG class subsets, we further classified them. Each XYi subset (i = 0, 1) was further classified into three subsets based on MN dinucleotides (M, N = A, T, C, G) content: XYi_MN0 (containing zero MN dinucleotides), XYi_ MN1 (containing one MN dinucleotide), and XYi_MN2 (containing two or more MN dinucleotides). Here, XY1 and XY2 subsets are referred to as XY1.

**Figure 2:**
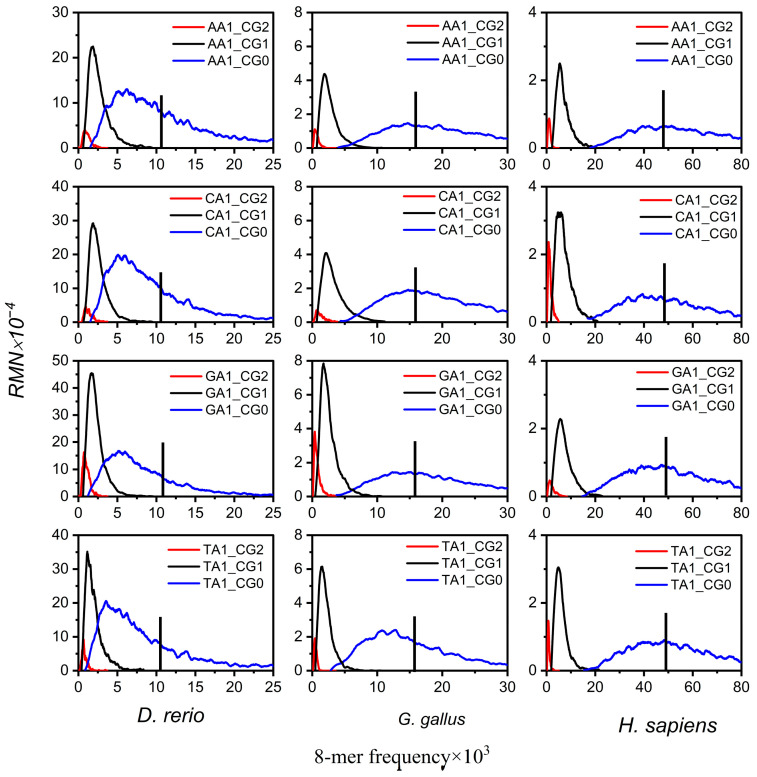
The 8-mer spectrum distributions of XY1_CGj subsets (X, Y = A, T, C, G, j = 0, 1, 2) for the three representative species’ genome sequences. The 8-mer spectrum distributions are shown for XY1_CG0 subsets (blue curve), XY1_GC1 subsets (black curve), and XY1_GC2 (red curve) subsets. The black vertical line is the random center.

First, we analyzed the non-CG class subsets spectra in mammalian genome sequences, all of which exhibit tri-modal distribution. *H. sapiens* was selected as the representative species (Figure 2). The 8-mer spectra of XYi_CG0, XYi_CG1, and XYi_CG2 form an independent unimodal distribution, and the distributions of the three peaks are separated. The most probable frequencies of XYi_CG1 and XYi_CG2 subsets are at low frequency and much lower than the random center. The most probable frequencies of the XYi_CG0 subset are near the random center. In addition, the spectrum of XYi_CG1 and XYi_CG2 subsets is much narrower than that of the XYi_CG0 subset, which indicates the usage of XYi_CG1 and XYi_CG2 8-mers is conservative. Except for the XYi_CGj subset, the 8-mer spectrum distributions of the other XYi_MNj subsets exhibit tri-modal, consistent with the distributions observed in XYi 8-mer spectra.

Second, the non-CG class subsets of spectra of other vertebrate genome sequences were analyzed. Additionally, their XYi 8-mer spectra are bimodal or unimodal; it was found that XYi_CG0, XYi_CG1, and XYi_CG2 8-mer spectra also formed an independent unimodal distribution, and the 8-mer spectrum distributions show higher conservatism in both XYi_CG1 and XYi_CG2 subsets compared to XYi_CG0 (Figure 2).

These results indicate that the distribution characteristics of 8-mer spectra in XYi_CG0, XYi_CG1, and XYi_CG2 subsets consistently display three properties of the CG-independent selection phenomenon. By analyzing the distribution characteristics of 8-mer spectra in 16 XYi subsets for vertebrate genome sequences, we found that their patterns in both CGi and XYi_CGj subsets vary substantially across species. To quantitatively characterize the distribution of CGi subsets, we calculated separability ($\delta_{CGi}$) and conservatism ($\beta_{CGi}$) for three CGi subsets in each species’ genome. Similarly, to quantitatively characterize the distribution of XYi_CGj subsets, we calculated separability ($\delta_{XYi\_CGj}$) and conservatism ($\beta_{XYi\_CGj}$) for the 90 XYi_CGj (i = 0, 1, j = 0, 1, 2) subsets within the non-CG class of each species. Overall, through the analysis of 8-mer distributions in the CGi/CGi_NMj subsets, we obtained a set of 186 class-level phylogenetic features, comprising 93 separability features and 93 conservatism features.

### 3.3. Order-Level Phylogenetic Features

**Figure 3:**
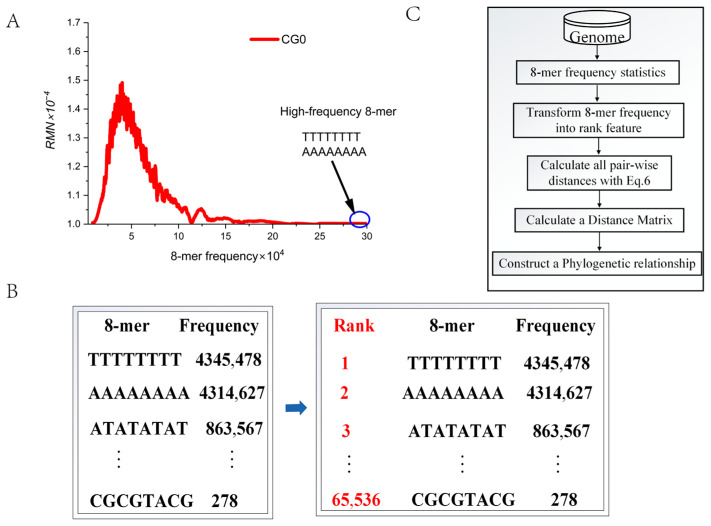
The rank values of 8-mers. (**A**) The 8-mer spectra of CG0 subsets in the human genome. (**B**) The conversion of 8-mer frequency data into rank information. (**C**) The algorithm flowchart.

According to the CG-independent selection phenomenon, the total 8-mer spectra of the genome sequence can be divided into three subsets: CG2, CG1, and CG0. Studies have shown that the 8-mer spectrum distributions of three CGi subsets do not conform to a normal distribution but follow a log-normal distribution and exhibit long-tail distribution characteristics [51]. When analyzing large-scale genome sequences, high-frequency 8-mers (for example, ‘TTTTTTTT’ and ‘AAAAAAAA’) are often concentrated in the long-tail region of the distribution (Figure 3A).

If the frequency of a specific 8-mer is abnormally high (exceeding the mean + 3 standard deviations), using its frequency directly as an evolutionary feature can pose problems, as this may skew the contribution from other evolution-related 8-mers. To address this issue, we convert frequency into rank information. The specific steps are as follows: assign a rank of 1 to the most frequent 8-mer, 2 to the next most frequent, and so on, with the lowest-frequency 8-mer receiving a rank of 65,536 (Figure 3B). Thus, we obtain 65,536 order-level phylogenetic features. We believe that rank information can more accurately capture subtle distance differences between genomes. In the following analysis, we will employ the algorithm flowchart (Figure 3C) to further validate the effectiveness of these 65,536 order-level phylogenetic features.

### 3.4. Phylogenetic Relationships with Class-Level Features

**Figure 4:**
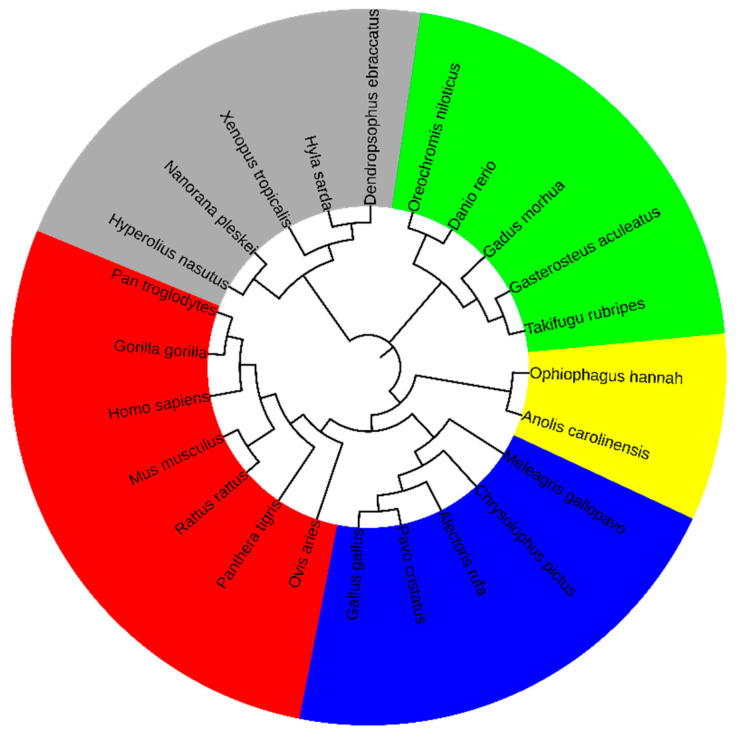
Phylogenetic relationship of vertebrates at the class level.

Based on the 8-mer spectra distribution characteristics of the CGi and XYi_CGj subsets in the genomic sequence, we identified the CG-independent selection phenomenon, from which features related to separability and conservatism were derived. To further validate that these features are closely associated with species evolution, we extracted a comprehensive set of 186 features, comprising 93 ($\delta_{i}$) and 93 ($\beta_{i}$). According to ‘Materials and Methods’, we calculated the distance matrix and constructed the phylogenetic relationships using Mega12 software, with the results shown in Figure 4.

Based on phylogenetic analysis, the species were categorized into five classes, with the following color background: Mammalia (red), Aves (blue), Lepidosauria (yellow), Amphibia (gray, and Actinopteri (green). Notably, the clustering pattern demonstrated strong phylogenetic consistency, with evolutionarily related species consistently grouped within the same clusters. This study demonstrates that the distribution characteristics of 8-mer spectra in the CGi and XYi_CGj subsets of vertebrate genome sequences can effectively capture the evolutionary information contained in the whole genome sequences. Furthermore, these results demonstrate that the 186 class-level phylogenetic features can clearly distinguish vertebrates at the class level.

### 3.5. Phylogenetic Relationships with Order-Level Features

#### 3.5.1. Phylogenetic Relationships of Mammalian Genome Sequences

**Figure 5:**
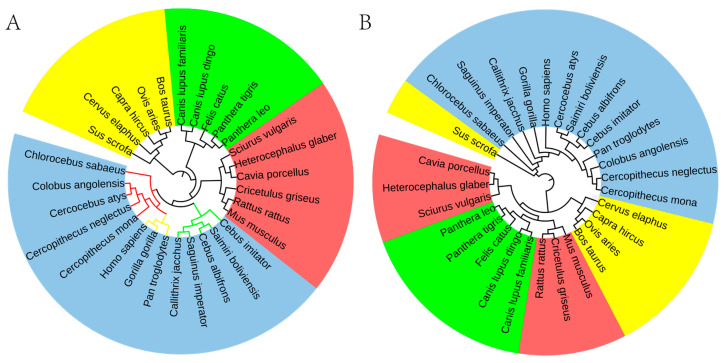
Phylogenetic relationships of mammals at the class level. (**A**) Phylogenetic relationships were constructed based on the rank information of 8-mer. (**B**) Phylogenetic relationships were constructed based on the FFP method.

To verify the reliability of the selected order-level phylogenetic features, we constructed phylogenetic relationships using the rank information of all 65,536 8-mers as features. According to Section 2 based on primate genome sequences, we used Mega12 software to calculate the distance matrix and construct the phylogenetic relationships, with the results shown in Figure 5.

Based on the rank information of 65,536 8-mers, the phylogenetic relationships we successfully constructed are highly consistent with those in known phylogenetic databases. Our phylogenetic analysis delineates four major mammalian orders: Primates (indigo), Rodentia (red), Artiodactyla (yellow), and Carnivora (green). Within the primate lineage, three family-level branches are identified. Notably, Anthropoids and Old-World monkeys form a sister clade with a close evolutionary relationship, distinct from the branch containing New World monkeys (Figure 5A).

These results collectively suggest the superior accuracy and stability of our method in constructing mammalian evolutionary relationships over traditional approaches. To support this, we conducted a comparative analysis with the classical FFP method. The results clearly demonstrate that the FFP method produces significant discrepancies from established phylogenetic conclusions when classifying the four mammalian orders. Specifically: (a) Sus scrofa, as an Artiodactyla species, was incorrectly clustered with Primates in the phylogenetic tree; (b) Rodentia were unreasonably divided into two evolutionary branches, one clustering with Carnivora and the other with Artiodactyla. (Figure 5B).

In the study of human evolution, Kasperski et al. [52] conducted a comprehensive analysis of evolutionary data across the following groups: monkeys (including tree shrews, prosimians, New World monkeys, and Old-World monkeys), other hominoids, and *H. sapiens*. Their results reveal distinct evolutionary distances between these groups, indicating that each taxon may be regulated by the separated genome attractors. Specifically, the distance factor between the attractor orbits of *H. sapiens* and other hominoids is 1.1, reflecting a relatively close evolutionary relationship. In contrast, the distance factors between *H. sapiens* and Old-World monkeys and New World monkeys are 12.4 and 17.8, respectively, demonstrating significant evolutionary divergence. The observed pattern of inter-group distance distribution in this study aligns with the trends identified in our own research.

#### 3.5.2. Phylogenetic Trees of Other Vertebrate Genome Sequences

**Figure 6:**
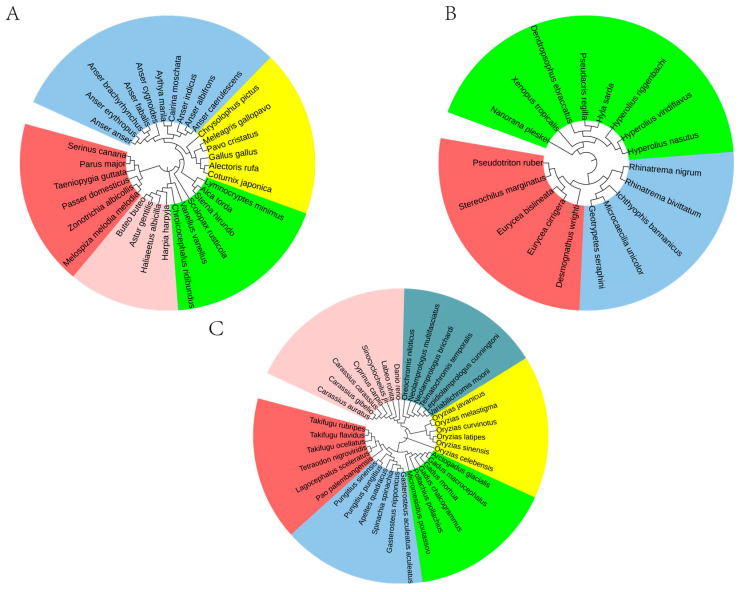
Phylogenetic relationships of other vertebrates at the order level. (**A**) Aves include Accipitriformes (red), Anseriformes (pink), Charadriiformes (green), Galliformes (yellow), and Passeriformes (indigo). (**B**) Amphibia include Anura (green), Caudata (red), and Gymnophiona (indigo). (**C**) Actinopteri include Beloniformes (yellow), Cichliformes (light green), Cypriniformes (pink), Gadiformes (green), Perciformes (indigo), and Tetraodontiformes (red).

To evaluate the general applicability of our method, we extended it to the genomic sequences of other vertebrate species. Phylogenetic relationships were inferred based on the rank information of 65,536 8-mers, with the results presented in Figure 6.

As shown in Figure 6A, our method clearly delineates major avian orders: Accipitriformes, Anseriformes, Charadriiformes, Galliformes, and Passeriformes as distinct monophyletic clades. Similarly, within the Actinopterygii class, six orders (Beloniformes, Cichliformes, Cypriniformes, Gadiformes, Perciformes, and Tetraodontiformes) are precisely resolved into six separate branches (Figure 6B). Likewise, the three amphibian orders (Anura, Caudata, and Gymnophiona) were also resolved as distinct clades with strong support (Figure 6C). These results demonstrate that our genomic order-level phylogenetic features based on 65,536 8-mers enable accurate species classification at the ordinal level.

For the three vertebrate groups examined, the inferred evolutionary relationships align with established findings [53,54,55,56,57], and the classification results are satisfactory. For instance, within the class Aves, Galliformes and Passeriformes exhibit a close evolutionary relationship, consistent with the conclusions of Stiller [53]. Similarly, within Actinopteri, Tetraodontiformes and Perciformes show a close affinity, further supporting the findings of Li [56]. These results confirm that the phylogenetic method developed in this study is applicable across species.

Figure 6 uncovers the evolutionary architecture of “separated genome attractors” through phylogenetic analysis of whole-genome data. The analysis demonstrates that major vertebrate lineages are organized into discrete genomic clusters (attractors) rather than forming a continuum in phylogenetic space. Within Aves, orders including Accipitriformes, Anseriformes, Charadriiformes, Galliformes, and Passeriformes form well-defined, non-overlapping clades. This pattern of phylogenetic segregation is similarly evident in Actinopterygii (exemplified by Cypriniformes, Perciformes, and Tetraodontiformes) and Amphibia (represented by Anura, Caudata, and Gymnophiona), where each order occupies a distinct and separated position within the phylogenetic framework. This consistent pattern of discrete clustering across diverse vertebrate classes provides compelling visual support for the separated genome attractor model, suggesting that each order has evolved to occupy and maintain a unique, stable region within the genomic adaptive landscape over macroevolutionary timescales.

## 4. Discussion

Previous work revealed that, among the 16 XY dinucleotide classifications, only the three CGi (i = 0, 1, 2) subsets within the CG classification meet all three properties of the CG-independent selection phenomenon. On this basis, we further analyzed the distribution characteristics of 8-mer spectra in non-CG class subsets. As genomic complexity increased during evolution, these distributions consistently transitioned from unimodal to multimodal, similar to the total 8-mer spectra, suggesting non-CG class 8-mer spectra contain more genomic evolutionary features. Thus, for each XYi (i = 0, 1) subset in the no-CG class 8-mer, we further partition it into XYi_MNj subsets, among which only the XY1_CGi/XY0_CGi (i = 0, 1, 2) subsets exhibit the CG-independent selection phenomenon. Based on the phenomenon of CG-independent selection, we identified a common characteristic in the spectrum distribution of the CGi, XY1_CGi, and XY0_CGi subsets: 8-mers containing CG are predominantly distributed in the low-frequency region of the spectrum, whereas those without CG are concentrated in the high-frequency region. This observation leads us to hypothesize that the 8-mers containing the CG dinucleotide are evolutionarily conserved functional motifs. Notably, in the *H. sapiens* genome, the CG2 subset demonstrates significant enrichment in CpG island regions, while the CG1 subset constitutes a core structural unit of nucleosomes [58]. These findings suggest that the CG-independent selection phenomenon has important guiding significance for mining evolutionary features of eukaryotic genome sequences.

**Figure 7:**
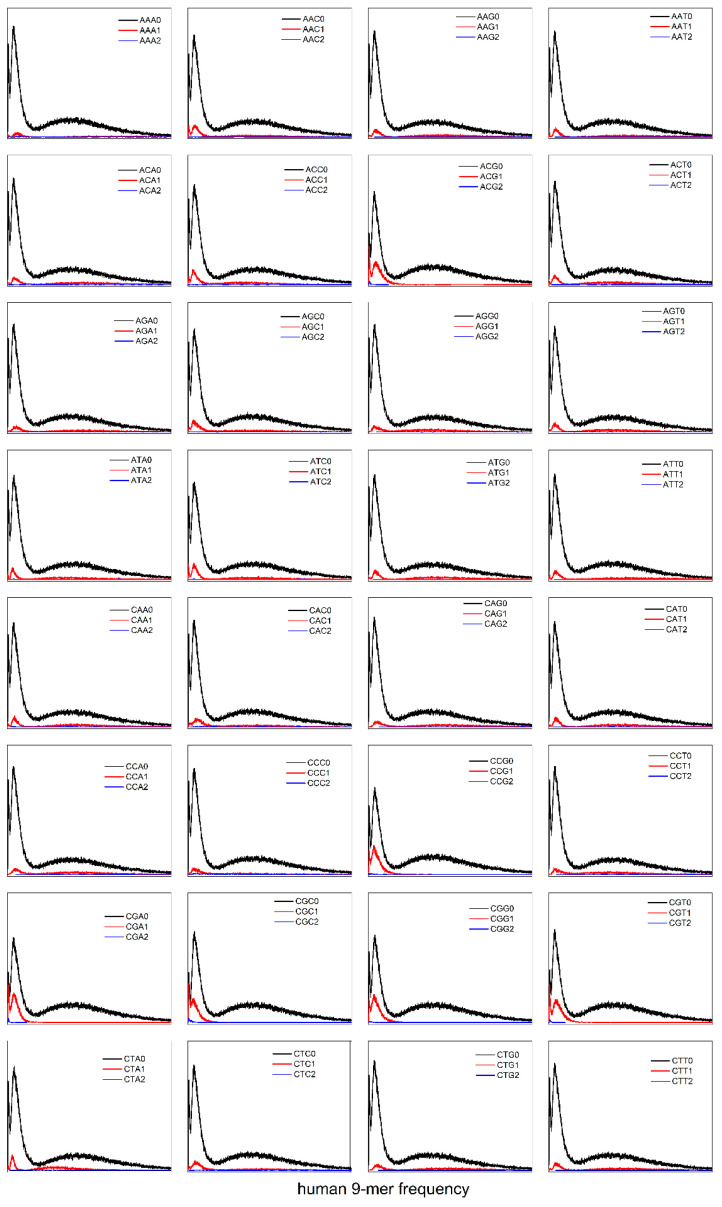
The 9-mer spectrum distributions of XYZ2, XYZ1, and XYZ0 subsets in the *H. sapiens* genome sequence (X, Y, Z = A, C, G, T).

Does the CG-independent selection phenomenon apply exclusively to the 8-mer subset spectrum within the XY dinucleotide classification? We extended the analysis of the CG-independent selection phenomenon from XY dinucleotide to XYZ trinucleotide k-mer spectra. To investigate the potential CG-independent selection phenomenon in XYZ trinucleotide k-mer spectra, we analyzed the *H. sapiens* genome as a representative species due to its distinct tri-modal distribution pattern. Using the XYZ trinucleotide classification method (“Methods” section), we characterized the distribution patterns of 9-mer spectra in 64 XYZi (i = 0, 1, 2) subsets (Figure 7). The analysis revealed that the distribution patterns of 9-mer spectra in 64 XYZi subsets fail to demonstrate independent unimodal distributions. Instead, each XYZi subset maintains a spectrum pattern consistent with the total 9-mer distribution (Figure 7), preserving the tri-modal distribution patterns. Significantly, the spectra of the CGXi/XCGi (X = A, T, C, G) subsets fail to demonstrate an independent unimodal distribution, thereby refuting the property of Evolutionary independence. Our results demonstrate that the CG-independent selection phenomenon is exclusively observed in XY dinucleotide classification motif subsets, but not in XYZ trinucleotide classification motif subsets.

Current phylogenomic studies employing k-mer-based methodologies face two persistent methodological constraints. First, constructing robust phylogenetic trees often requires filtering out high- or low-frequency k-mers, a process that inevitably discards genomic information and undermines the completeness of sequence representation. Second, simply using the frequency of k-mers as an evolutionary feature is inadequate, as it disrupts the balanced contribution of each evolutionarily relevant k-mer to phylogenetic inference. To overcome these challenges, this study introduces a novel ranking-based transformation approach. By transforming k-mer frequencies into rank-based features, we generate an ordinal feature profile. This approach circumvents the information loss associated with filtering while inherently equalizing the contribution of each k-mer through the ranking mechanism. As a result, our method preserves full genomic information while improving the robustness and reliability of phylogenetic inference.

Whole genome sequences provide a rich source of molecular data with great potential for revealing novel evolutionary insights. However, we currently lack mathematical models capable of capturing the heterogeneity of genome-wide evolution. Existing models are largely confined to single genes or specific genomic regions and thereby cannot integrate complex evolutionary information across the entire genome. It should be noted that phylogenetic relationships based on a single coding gene or gene set are called gene trees [38]. Thus, strictly speaking, gene trees can only represent evolutionary information of selected genes, but cannot represent the whole genome sequence evolutionary information of species [59,60]. Our research demonstrates that the 8-mer spectrum comprehensively captures whole-genome evolutionary information. Through comparative analysis of 8-mer spectrum distribution characteristics, we have established a powerful novel approach for constructing phylogenetic relationships at the whole genome level.

This study primarily focused on vertebrates and found that 186 features related to separability and conservatism can effectively construct phylogenetic relationships at the class level, while utilizing 65,536 rank features further enhances resolution, enabling fine-grained differentiation at the order level. In future work, we will extend this approach to invertebrates to systematically examine the cross-clade robustness and generality of the feature set, thereby investigating the uniformity and diversity of genomic 8-mer evolutionary patterns across different evolutionary scales. Genomic evolutionary features were categorized into macroevolutionary parameters (e.g., GC content, GC bias) and microevolutionary parameters (e.g., 65,536 ranking features). Subsequent research will integrate both types of metrics to reconstruct more reliable and higher-resolution phylogenetic relationships.

## 5. Conclusions

Based on the distribution characteristics of 8-mer spectra, we developed a novel dual-feature strategy for inferring phylogenetic relationships from genomic sequences. Firstly, we analyzed the spectrum distribution characteristics of 16 XYi subsets and identified the CG-independent selection phenomenon closely associated with species evolution. Based on this finding, we defined 186 class-level phylogenetic features, including 93 separability and 93 conservatism features. Secondly, leveraging the long-tail distribution of 8-mer spectra in the CGi subset, we derived 65,536 order-level phylogenetic features. To evaluate the proposed features, we tested them on multiple genome datasets spanning mammals, birds, Lepidosauria, amphibians, and fish. Results demonstrate that both class-level and order-level features serve as effective discriminators in genome sequence comparison. Further phylogenetic analysis revealed that trees constructed from class-level features resolve class-level relationships, whereas those built using order-level features achieve finer resolution at the order level. This study provides a new approach for constructing evolutionary relationships at the genomic level.

## Figures and Tables

**Table 1:** Number of species genome sequences.

Species	No.	Species	No.	Species	No.
Mammalia	29	Galliformes	6	Perciformes	6
Primates	13	Passeriformes	6	Tetraodontiformes	6
Rodentia	6	Lepidosauria	2	Amphibia	18
Artiodactyla	5	squamates	2	Anura	8
Carnivora	5	Actinopteri	37	Caudata	5
Aves	32	Beloniformes	6	Gymnophiona	5
Accipitriformes	4	Cichliformes	6		
Anseriformes	10	Cypriniformes	7		
Charadriiformes	6	Gadiformes	6		5

## Data Availability

Eukaryotic genome sequences and the corresponding annotation information were obtained from NCBI (https://www.ncbi.nlm.nih.gov/) and UCSC (http://genome.ucsc.edu/). The source code for calculating 8-mer frequencies is publicly available at https://github.com/guojunliu7/8MER (accessed on 26 December 2025). The program is implemented in Python 3.

## Supplementary Materials

The following supporting information can be downloaded at: https://www.mdpi.com/article/10.3390/genes17010039/s1, Table S1: Eukaryotic genomes and their classification.
